# Vacancies in the Dental Arch

**Published:** 1882-07

**Authors:** W. C. Wardlaw


### ARTICLE IV.

### *Vacancies in the Dental Arch.*

BY W. C. WARDLAW, D. D. S

A single gap in the dental arch is often the fruitful cause of many evils. Chewing upon the opposite side of the mouth is, perhaps, the most common and greatest evil consequent upon one such vacancy. Where there are several, occasioning a positive difficulty in the proper comminution of the food, by reason of the sufficient articulation of masticating surfaces, then dyspepsia, with all its train of ills, is apt to follow. I know of no minor (?) operation in dentistry from which more comfort and utility is to be derived, than the filling of these breaks in the continuity of the arch, and that dentist who devises the simplest and most feasible method of doing this, will be a public benefactor and a humanitarian.

It certainly is practical to fill the vacancy caused by the loss of one, two, and perhaps three teeth, by securing, through plugging, to the adjoining teeth, a bar of gold, to which has been attached by soldering, a porcelain crown or crowns. The crown can be so nicely adjusted to the gum as to look well and natural, and if properly done, will be very stable and durable. It is a *nice* operation, and requires care and forethought, as well as skill and taste.

I take, as my illustration of the crown to be supplied, the left upper first bicuspid. First, give attention to the formation of the cavities, which are to be prepared with more than usual care. They are supposed to be in the approximal surfaces of the canine and second bicuspid, and if no cavity of decay already exists, artificial mortises, or slots, will have to be cut in these positions. The thorough preparation of the cavities, and the taking of an accurate impression in plaster—not wax—of the parts, may be enough for one sitting, for both patient and operator. The entire operation would be too tedious, and this is a good half-way stopping place. Therefore fill the cavities temporarily with gutta-percha, or oxyphosphate, and dismiss the patient. From the impression, obtain a correct plaster model, upon which a large proportion of the work can be done without the necessity of the presence of the patient.

Having next carefully selected a crown—a cuspid crown would likely suit best—of proper shade, and ground to fit the gum nicely, solder on a gold backing. The backing may be prolonged a fourth of an inch, to rest upon the inside of the alveolar ridge This may be done by bending with the pliers, but really easier, by swaging up a small saddle to bestride the ridge, and to which the crown is to be soldered. This increased base not only adds greatly to the stability and permanency of the operation, but assists materially in steadying the crown and in keeping it in proper position during the operation of anchoring the cross-bar.

The next step is to make and attach to the backing of the crown a gold cross-bar of sufficient strength. This should be as long as the cavities in the teeth will allow, and should be so arranged relative to them that it will be supported on both sides, edges and ends, by the gold filling. Having so arranged all this by aid of the model, the two are to be soldered together, and all the crevices to be filled with solder or foil.

When the patient returns, the operation is to be resumed by preparations for keeping the cavities dry whilst they are being filled. For this purpose, I have used the wax ligature, passed twice around the tooth, but I find the rubber-dam better. It is not well to include both teeth in the same piece of rubber, for it is apt either to fold bunglingly in the way, or to exert a traction upon the crown so as to disturb its close adaptation to the gum. Begin, therefore, with the second bicuspid, and taking a small piece of rubber, cut a hole as near the edge—say one thirty-second of an inch—as will secure immunity from moisture. Use annealed foil, and anchor well the filling at the cervical wall, and build down squarely to where the upper edge of the bar will have to rest. Now comes the particular part of the operation—the filling of the space between the buccal wall of the cavity and the bar in position. For it must be remembered that it cannot be done well, and properly finished up, after the crown is anchored in place. Put the crown in place and fill the space between the bar and the palatine wall of the cavity with quick-setting oxyphosphate cement, and when it hardens, remove the crown. Slip in alongside the cement a matrix of wood or gold plate of the thickness of the bar, and continue to build down the filling in the cavity thus formed to the point to which the lower edge of the bar will come. Now carefully trim off, finish up and burnish with files, polishing tape, etc., so much of the filling as is inserted. Pursue the same course and proceed to the same point with the cavity in the canine, and remove the cement fillings. And now for the last time the crown is to be replaced, when it will be found that the bar will slip easily into place, maintained accurately in position by resting against the partially inserted fillings. The remainder is plain sailing. There will be no trouble in holding the crown in place with one hand and solidly filling the two simple cavities (easy of access from the palatine aspect) left between the bar and the palatine wall of the cavity, and thus the crown is immovably anchored in the two solid fillings surrounding the bar. The dressing off of the gold upon the palatine aspect is all that is left to be done, and is a simple matter, the access being easy.

And now you will hear your patient, say, "Doctor, that is so nice—feels so firm and natural. No one can detect it. I would four times rather have it than a plate."

As to the cost of such an operation; some people like to know the "cost" of things. The price should be somewhat "fancy," at least remunerative; for it is a *nice* operation, requiring the judgment, taste and skill not possessed by a second-class operator. A fair price, I think, would be the usual charge for two gold fillings and a single tooth upon a rubber plate.

A little reflection will enable one to modify these manipulations so as to meet the peculiar requirements of particular cases.

In conclusion, I would add that I think there are great possibilities involved in this principle, and I believe that the more it is practiced the greater it will extend the range of dentistry, particularly in the direction of increased masticatory powers—*Southern Dental Journal.*

